# Ghrelin attenuates the growth of HO-8910 ovarian cancer cells through the ERK pathway

**DOI:** 10.1590/1414-431X20155043

**Published:** 2016-02-02

**Authors:** R.X. Bai, W.P. Wang, P.W. Zhao, C.B. Li

**Affiliations:** 1 Department of Clinical Laboratory, Inner Mongolia People’s Hospital, Hohhot, China; 2 Graduate College, Inner Mongolia Medical University, Hohhot, China; 3 Microbiology and Immunology Laboratory, School of Basic Medical Science, Inner Mongolia Medical University, Hohhot, China; 4 School of Basic Medical Science, Inner Mongolia Medical University, Hohhot, China

**Keywords:** Ghrelin, MAPKs, HO-8910, Apoptosis

## Abstract

Ovarian cancer is one of the most common causes of death from gynecologic tumors and is an important public health issue. Ghrelin is a recently discovered bioactive peptide that acts as a natural endogenous ligand of the growth hormone secretagogue receptor (GHSR). Several studies have identified the protective effects of ghrelin on the mammalian reproductive system. However, little research has been done on the effects of ghrelin on ovarian cancer cells, and the underlying mechanisms of these effects. We sought to understand the potential involvement of mitogen-activated protein kinases (MAPKs) in ghrelin-mediated inhibition of growth of the ovarian line HO-8910. We applied different concentrations of ghrelin and an inhibitor of the ghrelin receptor (D-Lys3-GHRP-6) to HO-8910 cells and observed the growth rate of cells and changes in phosphorylation of the MAPKs ERK1/2, JNK and p38. We discovered that ghrelin-induced apoptosis of HO-8910 cells was though phosphorylated ERK1/2, and that this phosphorylation (as well as p90^rsk^ phosphorylation) was mediated by the GHSR. The ERK1/2 pathway is known to play an essential part in the ghrelin-mediated apoptosis of HO-8910 cells. Hence, our study suggests that ghrelin inhibits the growth of HO-8910 cells primarily through the GHSR/ERK pathway.

## Introduction

Every year more than 225,000 women are diagnosed with ovarian cancer and an estimated 140,000 deaths are caused by ovarian cancer worldwide (1). Five-year survival for patients with ovarian cancer is 92% if the tumor is detected at an early, localized stage. However, early diagnosis is rare; 85% of ovarian cancers are detected at a more advanced stage because symptoms are easily missed until the disease has spread to other body regions (2). Thus, the overall five-year survival for all women diagnosed with epithelial ovarian carcinoma is only 44% (2,3). Because of the overall high mortality of ovarian cancer, women at increased risk may be counseled to undergo prophylactic (and possibly unnecessary) oophorectomy (4).

Ghrelin is an endogenous ligand for the growth hormone secretagogue receptor (GHSR). It is a 28-amino acid peptide produced from a pre-prohormone with a length of 117 amino acids. The mature form of ghrelin can undergo several post-translational modifications, including addition of a fatty-acid chain (n-octanoic acid) to the serine 3 residue (5). Ghrelin has been shown to stimulate secretion of growth hormone, and to have orexigenic and adipogenic effects (6). Most ghrelin is produced in the stomach by a distinct group of endocrine cells located within the gastric oxyntic mucosa (7,8), with smaller amounts produced by other organs. Small amounts of ghrelin have also been observed elsewhere in the gastrointestinal tract and pancreas, and its activity influences the metabolism of glucose and lipids.

This peptide also has less well-understood roles in other tissues and organs. Ghrelin expression has been reported at low levels in the brain, pituitary gland, kidneys, and thyroid gland, as well as in several areas of the reproductive system, including the placenta, testes, and ovaries (9). A recent report describes ghrelin expression at mRNA and peptide levels in the ovaries of adult rats. mRNA levels of ghrelin in rat ovaries were monitored throughout the estrous cycle to better understand physiologic regulation of expression of the ghrelin gene in ovaries (10). Despite persistent expression of the signal throughout all stages of the estrous cycle, mRNA levels of ghrelin change in a cyclic fashion, with lowest expression occurring in the proestrus phase, and maximum values in the diestrous phase (11).

In many (though not all) normal tissues, ghrelin stimulates cell proliferation and protects against apoptosis (7). In contrast, there are numerous reports of ghrelin-mediated inhibition of the growth of cancer cells (9), including the ovarian cancer cells known as HO-8910 (2). There are conflicting reports describing positive and negative effects of ghrelin and other growth hormone-stimulating molecules on the growth of tumor cells *in vitro* (12,13). The activity of several signaling pathways, including mitogen-activated protein kinase (MAPK) pathways, have been implicated in these processes. We investigated if ghrelin exerts its inhibitory effects on HO-8910 cells through GHSR activation and the downstream activity of MAPKs.

## Material and Methods

Unless specified otherwise, all chemicals and reagents were purchased from Sigma-Aldrich (USA). Antibodies against IgG, glyceraldehyde-3-phosphate dehydrogenase (GAPDH), ERK1/2, JNK, p90rsk, phospho-ERK1/2, phospho-JNK and phospho-p90rsk1 (Ser380) were purchased from Millipore (USA). Unless specified otherwise, culture of the ovarian line HO-8910 (Chinese Academy of Sciences, China) took place at 38.5°C with 5% CO_2_ under humidified air. The HO-8910 cell line is derived from a 51-year-old Chinese patient with ovarian cancer and ascites in 1994.

### RNA extraction and reverse-transcription-polymerase chain reaction (RT-PCR)

Total RNA was isolated from HO-8910 cells using an RNeasy kit (Qiagen, Germany). RNA samples were treated with RNase-free DNase I to remove contamination of genomic DNA. RNA content of samples was too low to be quantified accurately by spectrometry. Thus, 6.5-µL RNA aliquots were converted to cDNA by reverse transcription, then amplified (Takara Bio, Japan). PCR primers for the ghrelin receptor were: sense, 5′-TCTTCCTTCCTGTCTTCTGTC-3′; antisense, 5′-AGTCTGAACACTGCCACC-3′ (14).

### 3-(4,5-dimethylthiazol-2-yl)-2,5-diphenyltetrazolium bromide (MTT) assay

Initially, cells were grown in 96-well plates (1×10^3^ cells/well) with ghrelin and D-Lys3-GHRP-6. Control cells were switched from RPMI1640 to Dulbecco’s modified Eagle’s medium (DMEM) containing 0.1% dimethyl sulfoxide (DMSO). At 12, 24, 36, 48, 60 and 72 h after treatment with ghrelin and D-Lys3-GHRP-6, 20 µL of MTT was added to each well to a final concentration of 0.5%. After 4 h incubation at 37°C in the dark, 150 µL DMSO was added to each well for 10 min to dissolve formazan crystals. Absorbance was measured using a microplate reader (ELx800; BioTek, USA) at 490 nm. Experiments were repeated three times. Viability of ghrelin- and D-Lys3-GHRP-6-treated cells was expressed as the percentage of population growth plus standard error of the mean relative to that of untreated control cells. Cell death caused by ghrelin and D-Lys3-GHRP-6 was calculated as a percentage of inhibition using the following formula: Percent inhibition = (1 - mean experimental absorbance/mean control absorbance) ×100.

### Assay to determine effective concentrations of ghrelin and D-Lys3-GHRP-6 (ghrelin receptor inhibitor)

Ghrelin was added to HO-8910 growth media to final concentrations of 121, 152, 182, 212, and 242 nM, cells were cultured for 12, 24, 36, 48, 60 and 72 h, and then the growth of HO-8910 cells was analyzed. Once the optimum ghrelin concentration and treatment duration to achieve inhibition were determined, this treatment was repeated with addition of D-Lys3-GHRP-6 to final concentrations of 10^-8^, 10^-9^, 10^-10^, and 10^-11^ mg/mL. HO-8910 cells were then cultured for 12, 24, 36, 48, 60 and 72 h, and their growth analyzed.

### Western blotting

HO-8910 cells were homogenized and proteins separated by electrophoresis on 8-12% sodium dodecyl sulfate/polyacrylamide gels, and then transferred to immunoblot nitrocellulose membranes. Membranes were blocked for 30 min at room temperature with phosphate-buffered saline (PBS) containing 5% fat-free milk and 0.1% Tween 20. Then, membranes were incubated with primary anti-Rac1 antibody for ≥1 h at room temperature, or overnight at 4°C. Then, membranes were washed thrice with PBS containing 0.1% Tween 20, incubated with peroxidase-conjugated secondary antibodies, and developed using ECL reagent (Pierce, USA).

### siRNA design

RNA interference was used to silence expression of ERK1/2 in HO-8910 cells. ERK1/2-siRNA (TGAATTGTATCATCAACAT) was synthesized by Gene Pharma Biotechnology (China).

### Transfection of siRNA

siRNA transfection was conducted using lipofectamine according to the protocol supplied by Invitrogen (USA). Briefly, 1×10^5^ cells were seeded onto six-well plates containing antibiotic-free medium and incubated overnight. For each well, 5 µL siRNA was mixed with 125 µL Opti-MEM I. The mixture was combined with a solution of 5 µL lipofectamine in 125 µL Opti-MEM I. After 20 min at room temperature, the mixture was applied to cells in an appropriate volume of Opti-MEM I to achieve a final concentration of 100 nM for each siRNA. Negative control group was transfected without siRNA. After incubation for 6 h at 37°C, RPMI1640 supplemented with serum was added to wells. Cells were cultured for an additional 24 h at 37°C before analyses.

## Results

### GHS-R expression in HO-8910 cells

**Figure 1:**
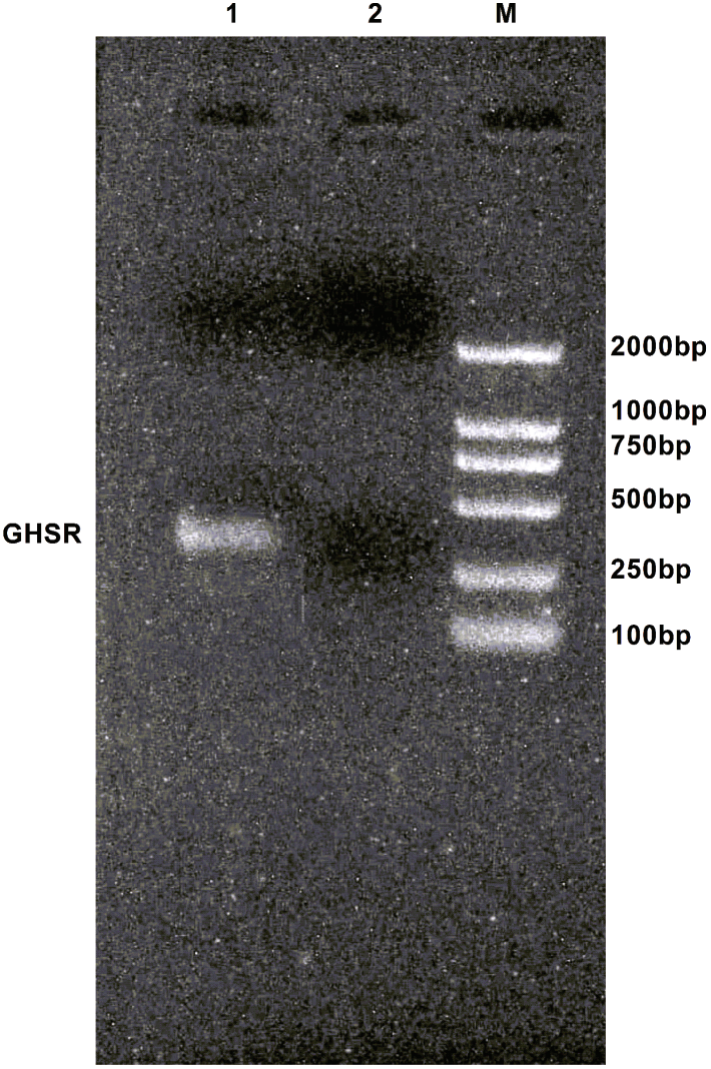
Expression of GHSR mRNA in HO-8910 cells by RT-PCR. 1: sample; 2: water blank; M: DNA Marker DL 2000.

RT-PCR was used to detect expression of *GHSR* mRNA in HO-8910 cells. The *GHSR* (348 bp) was expressed at a high level in HO-8910 cells (Figure 1).

### Optimal concentrations and timing of treatment of ghrelin and D-Lys3-GHRP-6 on HO-8910 cells

The MTT assay was used to assess the growth and viability of HO-8910 cells after treatment with varying concentrations of ghrelin, and to determine the appropriate duration of treatment to achieve the desired level of growth inhibition.

**Figure 2:**
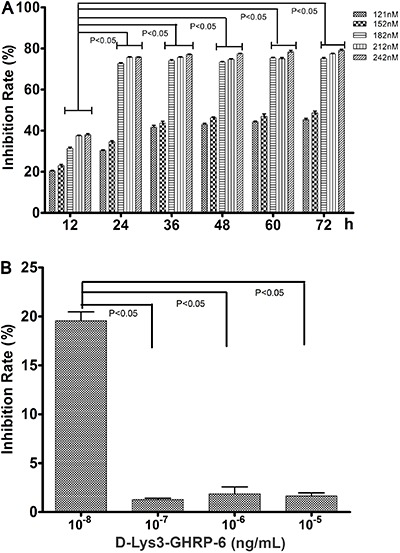
Growth of HO-8910 cells after treatment with ghrelin and with ghrelin plus D-Lys3-GHRP-6. *A*, Inhibition of growth 12, 24, 36, 48, 60 and 72 h after treatment with increasing concentrations of ghrelin. Data are reported as means±SD (n=5). *B*, Effect of D-Lys3-GHRP-6 treatment on cell growth after ghrelin treatment. One-way ANOVA was used for statistical analysis.

Ghrelin was added to HO-8910 media to final concentrations of 121, 152, 182, 212 and 242 nM. Numbers of viable cells were assessed at 12, 24, 36, 48, 60 and 72 h (Figure 2A). The optimal concentration and duration of ghrelin treatment for HO-8910 cells was found to be 182 nM and 24 h, respectively.

Next, we determined the D-Lys3-GHRP-6 concentration needed to prevent the growth inhibition caused by 182 nM ghrelin in HO-8910 cells. HO-8910 cells that had been treated with 182 nM ghrelin were treated with D-Lys3-GHRP-6 (10^-5^, 10^-6^, 10^-7^, 10^-8^ ng/mL) and cell numbers evaluated at 24 h (Figure 2B). We found that 10^-6^ ng/mL D-Lys3-GHRP-6 could inhibit the growth inhibition caused by 182 nM ghrelin.

### Ghrelin inhibited the growth of HO-8910 cells *via* the ERK1/2 pathway

**Figure 3:**
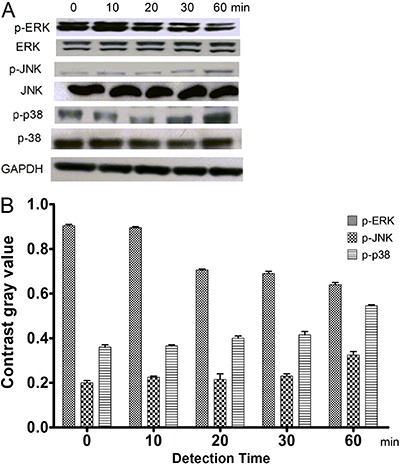
Effects of 182 nM ghrelin on mitogen-activated protein kinase (MAPK) activation in HO-8910 cells. *A*, Expression of total and phosphorylated ERK1/2, JNKs, and p38 proteins. *B*, Contrast gray value of the phosphorylation of ERK1/2, JNKs and p38 based on Western blotting.

To ascertain which pathway has a key role in ghrelin-mediated inhibition of the growth of HO-8910 cells, the phosphorylation status of the MAPKs ERK1/2, JNK and p38 was detected at 0, 10, 20, 30 and 60 min (Figure 3) after treatment with 182 nM ghrelin. A decrease in ERK1/2 phosphorylation was greater than that observed for JNK or p38 after 20 min.

**Figure 4:**
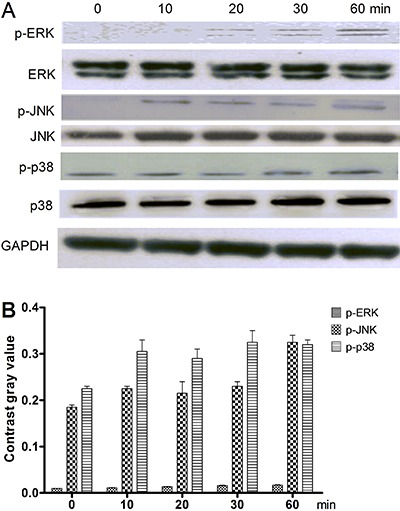
Effects of an inhibitor of the ghrelin receptor (D-Lys3-GHRP-6) on ghrelin-mediated MAPK activation in HO-8910 cells. *A*, Expression of total and phosphorylated ERK1/2, JNKs, and p38 proteins at 0, 10, 20, 30 and 60 min. *B*, Contrast gray value of the phosphorylation of ERK1/2, JNKs and p38 based on Western blotting.

When this treatment was repeated with addition of 10^-9^ mg/mL D-Lys3-GHRP-6, ERK1/2 phosphorylation was reduced compared with treatment with ghrelin alone. The phosphorylation status of JNK and p38 were not changed significantly (Figure 4).

**Figure 5:**
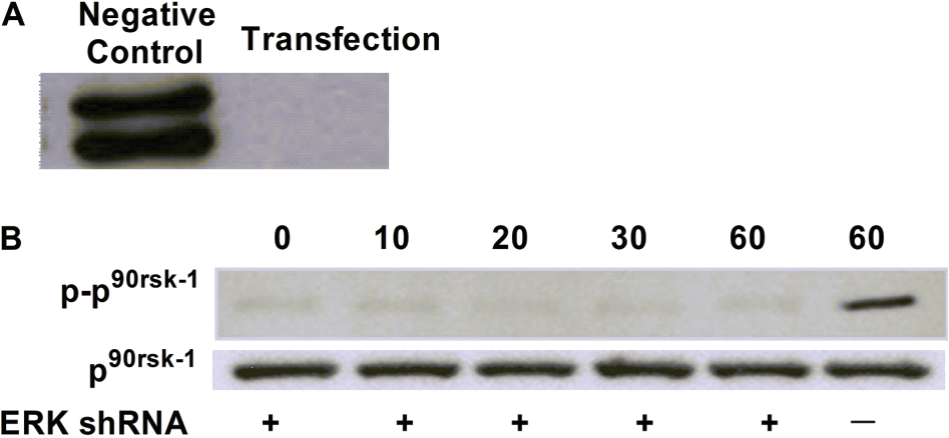
Effects siRNA knockdown of ERK1/2 on ERK1/2, p^90rsk^ and phosphorylation of p^90rsk^. *A*, Expression of ERK1/2 after ERK1/2 knockdown. *B*, Expression of p^90rsk^ and phospho-p^90rsk^ at different times after knockdown (0, 10, 20, 30, 60 min).

This result suggested that ERK1/2 has a key role in the ability of ghrelin to block the growth of HO-8910 cells. To verify this finding, siRNA specific for ERK1/2 was used to knock down ERK1/2 expression (Figure 5). Also, the phosphorylation of p^90rsk^ (p-p^90rsk^) was low (Figure 5).

**Figure 6:**
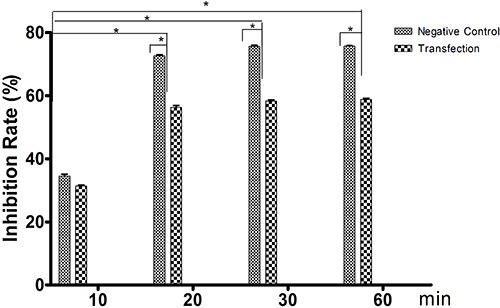
Prevention of ghrelin-mediated growth inhibition in HO-8910 cells after knockdown by siRNA of ERK1/2. *P<0.05 (one-way ANOVA).

After reduction of *ERK1/*2 by siRNA knockdown, ghrelin (182 nM) was no longer able to reduce the growth of HO-8910 cells (Figure 6).

## Discussion

Ghrelin is produced predominantly by the stomach, though smaller amounts are produced by the bowel, pancreas, pituitary, kidneys, and placenta. The GHSR is a typical G protein-coupled seven-transmembrane receptor (15). Several studies have identified the protective effects of ghrelin (4,16 17 18), but little is known about the effects of ghrelin on ovarian cancer cells or their mechanism of action. We found that the mechanism through which ghrelin inhibits the growth of HO-8910 cells involves the ERK1/2 branch of MAPK pathways. Maximum inhibition of the growth of HO-8910 cells was achieved using 182 nM ghrelin for 24 h. We also demonstrated that this effect of ghrelin was mediated through its receptor, the GHSR (19), which were shown to be expressed at high levels in HO-8910 cells. Experiments in which the GHSR inhibitor D-Lys3-GHRP-6 (10^-6^ ng/mL) blocked ghrelin-mediated growth inhibition provided further support for the role of the GHSR.

To gain further insight into the mechanism by which ghrelin inhibits growth of HO-8910 cells, we evaluated the activity of signaling pathways downstream of the GHSR. MAPKs are a family of serine/threonine kinases that includes ERK, JNK and p38. These kinases are involved primarily in activation of the nuclear transcription factors that control the proliferation, differentiation and apoptosis of cells (20). Our study suggests that ghrelin inhibits the growth of HO-8910 cells *via* the ERK signaling pathway, and not through activation of JNK or p38. We found that 20–60 min of ghrelin treatment was required to inhibit ERK phosphorylation, so stimulation was time-dependent. Furthermore, GHSR blockade by chemical inhibition and silencing of ERK by siRNA suppressed ghrelin-mediated inhibition of the growth of HO-8910 cells.

Overall, our study suggests that the ghrelin/GHSR signaling pathway attenuates the growth of HO-8910 cells mainly through an ERK-dependent pathway. Thus, ghrelin could be a target for ovarian cancer therapy. Despite this promising finding, further study is necessary before clinical application is considered.
